# Towards comprehensive assessment of mitral regurgitation using cardiovascular magnetic resonance

**DOI:** 10.1186/1532-429X-10-61

**Published:** 2008-12-22

**Authors:** KM John Chan, Ricardo Wage, Karen Symmonds, Shelley Rahman-Haley, Raad H Mohiaddin, David N Firmin, John R Pepper, Dudley J Pennell, Philip J Kilner

**Affiliations:** 1 Cardiovascular Magnetic Resonance Unit, Royal Brompton and Harefield NHS Trust, Royal Brompton Hospital, Sydney Street, London SW3 6NP, UK; 2 Department of Cardiothoracic Surgery, Royal Brompton and Harefield NHS Trust, Royal Brompton Hospital, Sydney Street, London SW3 6NP, UK; 3 Department of Cardiology, Royal Brompton and Harefield NHS Trust, Harefield Hospital, Hill End Road, Harefield, Middlesex UB9 6JH, UK; 4 National Heart and Lung Institute, Imperial College London, Royal Brompton Hospital, London SW3 6NP, UK

## Abstract

Cardiovascular magnetic resonance (CMR) is increasingly used to assess patients with mitral regurgitation. Its advantages include quantitative determination of ventricular volumes and function and the mitral regurgitant fraction, and in ischemic mitral regurgitation, regional myocardial function and viability. In addition to these, identification of leaflet prolapse or restriction is necessary when valve repair is contemplated. We describe a systematic approach to the evaluation of mitral regurgitation using CMR which we have used in 149 patients with varying etiologies and severity of regurgitation over a 15 month period.

Following standard ventricular cine acquisitions, including 2, 3 and 4 chamber long axis views and a short axis stack for biventricular function, we image movements of all parts of the mitral leaflets using a contiguous stack of oblique long axis cines aligned orthogonal to the central part of the line of coaptation. The 8–10 slices in the stack, orientated approximately parallel to a 3-chamber view, are acquired sequentially from the superior (antero-lateral) mitral commissure to the inferior (postero-medial) commissure, visualising each apposing pair of anterior and posterior leaflet scallops in turn (A1-P1, A2-P2 and A3-P3). We use balanced steady state free precession imaging at 1.5 Tesla, slice thickness 5 mm, with no inter-slice gaps. Where the para-commissural coaptation lines curve relative to the central region, two further oblique cines are acquired orthogonal to the line of coaptation adjacent to each commissure. To quantify mitral regurgitation, we use phase contrast velocity mapping to measure aortic outflow, subtracting this from the left ventricular stroke volume to calculate the mitral regurgitant volume which, when divided by the left ventricular stroke volume, gives the mitral regurgitant fraction. In patients with ischemic mitral regurgitation, we further assess regional left ventricular function and, with late gadolinium enhancement, myocardial viability.

Comprehensive assessment of mitral regurgitation using CMR is feasible and enables determination of mitral regurgitation severity, associated leaflet prolapse or restriction, ventricular function and viability in a single examination and is now routinely performed at our centre. The mitral valve stack of images is particularly useful and easy to acquire.

## Background

Mitral regurgitation is a relatively common and important heart valve lesion in clinical practice, and adequate assessment is fundamental to decisions on management. Patients with less than severe mitral regurgitation are generally managed conservatively with medical treatment whereas patients with severe mitral regurgitation are considered for surgery, especially if the left ventricle (LV) shows signs of dilation or impairment of function, or if the patient is symptomatic [1]. Increasingly, asymptomatic patients with relatively normal LV function and only mild LV dilatation are considered for mitral valve repair rather than replacement as this has been shown to improve long term outcome and the risk of surgery is low [1]. Assessment of the severity of regurgitation, the dysfunction responsible for it (leaflet prolapse or restriction), the etiology of the condition (degenerative, ischemic, rheumatic, cardiomyopathy), and LV size and function, are each important. Additional investigations may be indicated, particularly in ischemic mitral regurgitation, where evaluation of coronary artery lesions, myocardial viability and contractile reserve are relevant.

Echocardiography is the recognised investigation of choice for mitral regurgitation [1]. However, the technique is operator dependent and may not always give optimal diagnostic views of mitral valve dysfunction. Transesophageal echocardiography, with 3 dimensional visualisation if available, generally gives a greater overall assessment of mitral valve dysfunction and the lesions responsible for it, but is also operator dependent, semi-invasive and usually requires patient sedation.

Although cardiovascular magnetic resonance (CMR) is not as widely used in the assessment of mitral regurgitation, it has significant strengths. These include accurate determination of left as well as right ventricular volumes and function [2,3], measurements of aortic flow volume, and in ischemic mitral regurgitation, comprehensive assessment of regional myocardial function and viability [2,4]. Although quantification of the severity of mitral regurgitation using CMR is well established [3,5-7], assessment of the valve dysfunction responsible for it requires acquisitions planned with respect to mitral structure and movement. Dedicated cine imaging planes in addition to the standard long axis views are needed to assess movements of each scallop of each leaflet of the mitral valve. When used optimally, CMR can complement echocardiography in the assessment of mitral regurgitation, especially in patients in whom transthoracic echocardiography has not provided adequate information.

### Mitral valve anatomy

**Figure 1:**
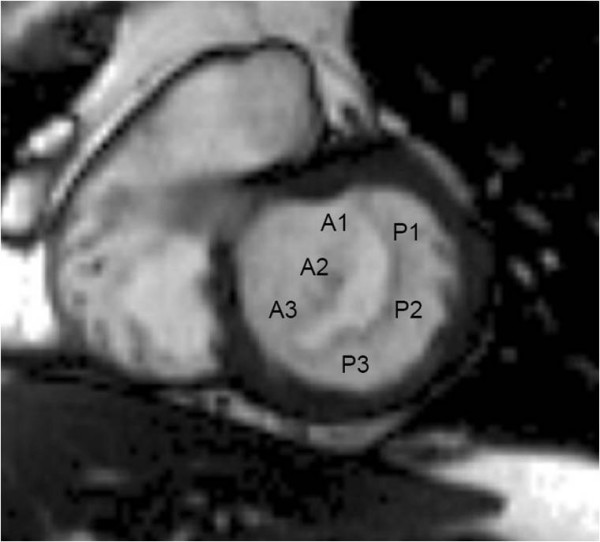
**Anatomy of the mitral valve**. CMR short axis view of the mitral valve from a basal short axis slice showing its two leaflets (anterior and posterior) and the three scallops of each leaflet (A1, A2 and A3 in the anterior leaflet, P1, P2 and P3 in the posterior leaflet). The mitral valve is viewed from the LV looking towards the left atrium.

An understanding of the anatomy of the mitral valve is required for optimal imaging. The valve consists of two leaflets: anterior (or antero-septal) and posterior (or postero-lateral). Each leaflet is divided, from top to bottom, into 3 scallops: A1, A2 and A3 of the anterior leaflet and P1, P2 and P3 of the posterior leaflet (Fig 1) [8]. The mitral valve leaflets are inserted at the base of the LV at the annulus, and their free edges are attached by multiple chordae tendinae to the papillary muscles. Adequate coaptation of the free edges of the valve leaflets is necessary to achieve valve competency. Degenerative mitral valve disease or myxomatous degeneration of the leaflets with redundant or thickened leaflet tissue, or ruptured or elongated chordae or papillary muscles, typically results in leaflet prolapse and regurgitation. The prolapse may involve the entire leaflet or, more commonly, only one or two scallops. It is important to determine both the location and extent of the leaflet prolapse e.g. posterior leaflet prolapse at P2 and P3, or anterior leaflet prolapse at A2, etc. This is necessary to determine the type of surgery most likely to be successful (mitral valve repair or replacement) and the extent of surgical repair needed. Mitral regurgitation from prolapse of P2, for example, is generally amenable to surgical repair whereas anterior leaflet prolapse is generally more challenging.

## Mitral imaging technique

**Figure 2:**
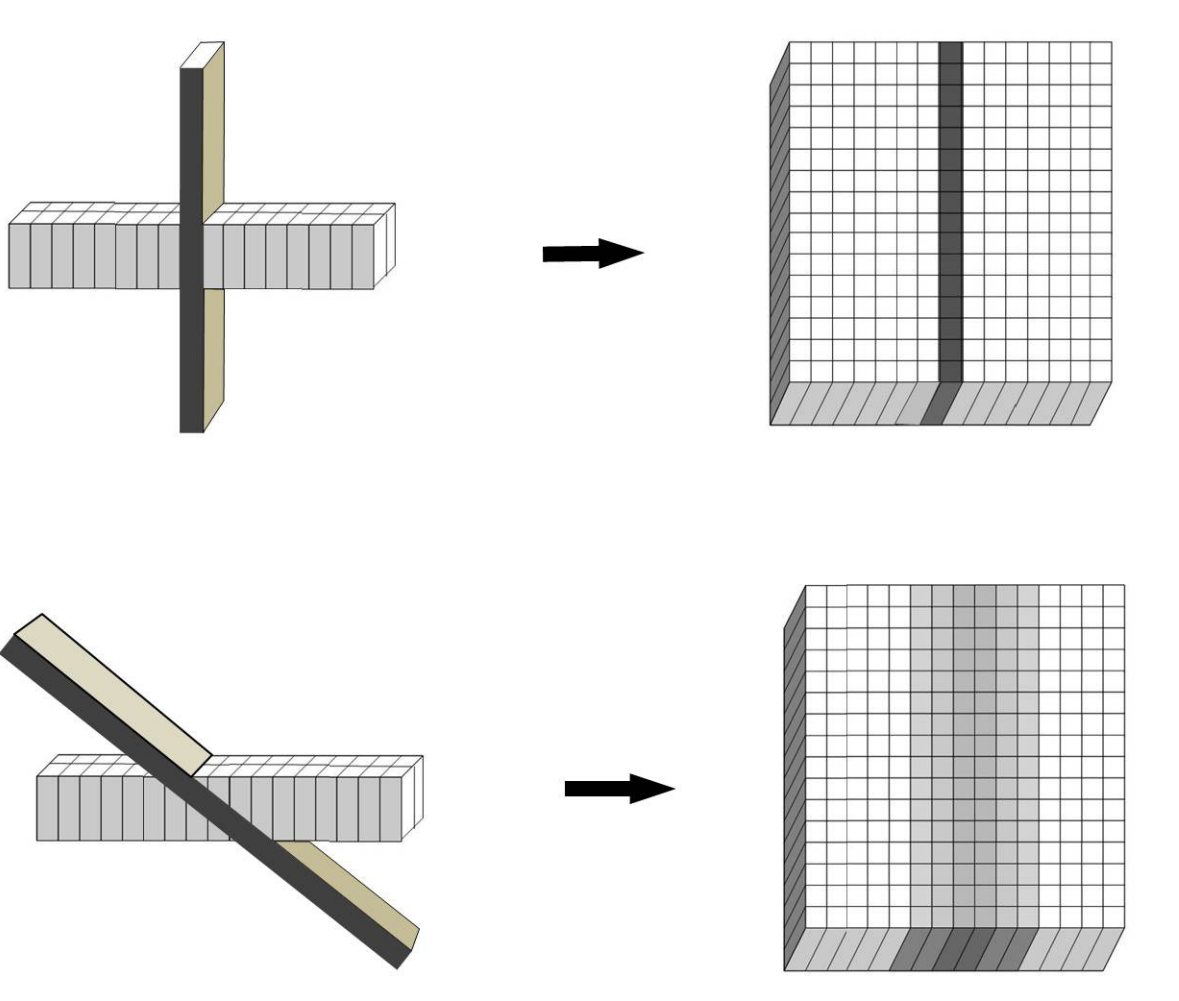
**Imaging a thin structure or boundary using a relatively thick slice**. Because the voxels that contribute to a CMR cine image are elongated, their length being the thickness of the slice, orthogonal orientation of the slice relative to a thin structure or boundary depicts the structure more clearly than oblique orientation. This principle is fundamental to the strategies described in this paper for the imaging of the mitral leaflets and associated regurgitant jets.

**Figure 3:**
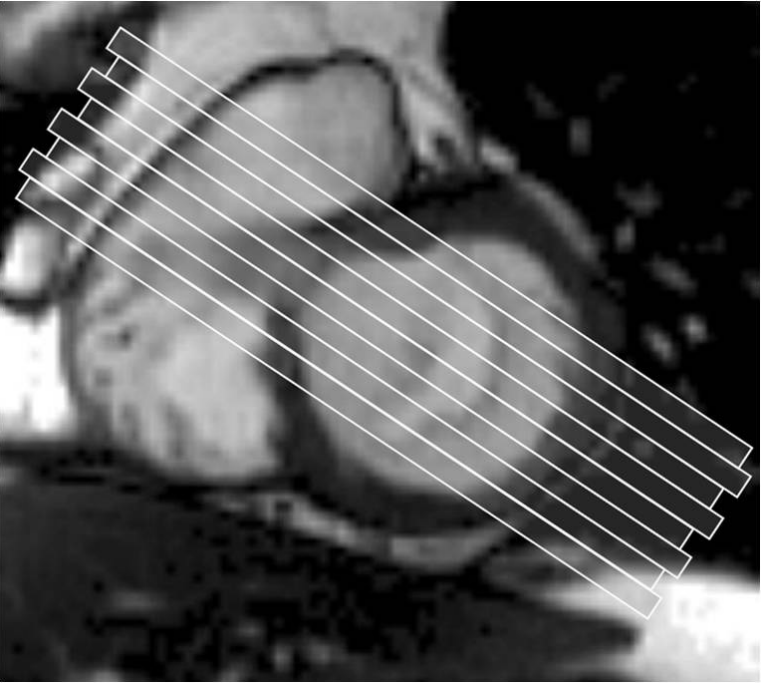
**Slices taken across the mitral valve**. 5 mm thick slices are taken starting from the superior (antero-lateral) commissure (A1-P1) and moving towards the inferior (postero-medial) commissure (A3-P3) at 5 mm intervals. The orientation of the slice is parallel to the LVOT slice.

**Figure 4:**
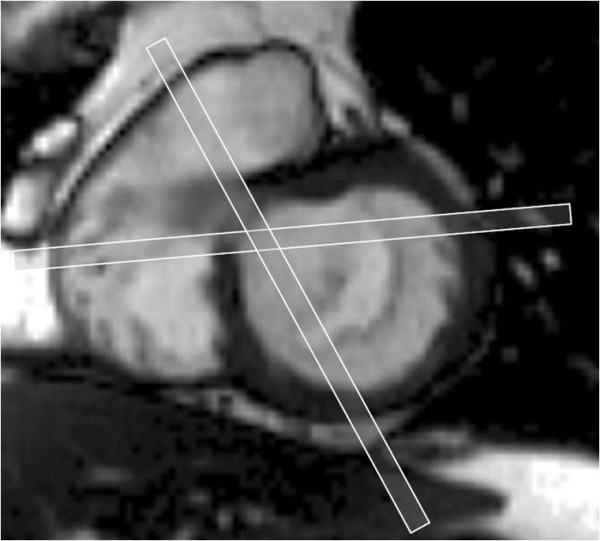
**Additional imaging slices at each end of the mitral valve**. A further pair of slices orthogonal to the coaptation plane of the valve leaflets is taken at the commissures at each end of the mitral valve (A1-P1 and A3-P3) to better visualise these scallops.

**Figure 5:**
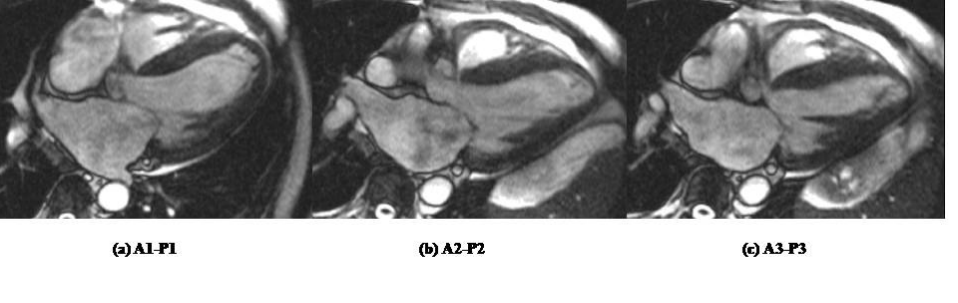
**Images obtained of each scallop of the mitral valve**. Each scallop of both mitral valve leaflets is clearly visualised: (a) A1-P1, (b) A2-P2, (c) A3-P3. Moderate centrally directed mitral regurgitation is seen most marked at (b) A2-P2 due to leaflet restriction following myocardial infarction.

We use balanced steady state free precession (bSSFP) end-expiratory breath hold cines, retrospective ECG gating, temporal resolution of 20–30 ms, echo time 1.13 ms, in-plane pixel size 1.7 × 1.7 mm, flip angle 80 degrees, FOV read 320 mm, FOV phase 220 mm, base resolution 192 pixels, and acquisition time 16 heart beats. Following standard imaging sequences, including cines of 2, 3 and 4 chamber long axis views and a short axis stack (starting at the mitral annulus and continuing to the LV apex using a slice thickness of 8 mm and a 2 mm gap), the mitral valve is thoroughly imaged. A principle that is fundamental to CMR cine imaging of thin structures or boundaries such as those of mitral leaflets or jets is illustrated (Fig 2). A basal short axis slice which shows the mitral valve is selected (Fig 1). From this, a contiguous stack of oblique slices are aligned orthogonal to the central part of the line of coaptation, orientated approximately parallel to the 3-chamber LVOT long axis plane. The stack of cines acquired starts from the superior (antero-lateral) commissure adjacent to A1-P1 and progresses towards the inferior (postero-medial) commissure adjacent to A3-P3 using a slice thickness of 5 mm and no inter-slice gap (Fig 3). Typically, 2–3 slices pass through each of the valve scallops (A1-P1, A2-P2, A3-P3). A further pair of oblique slices is acquired orthogonal to the oblique line of leaflet coaptation at each end of the valve adjacent to the commissures (across A1-P1 and A3-P3) (Fig 4). These additional slices help to visualise the leaflets and their function in the commissural region where their orientation is oblique to the initial cine stack (Figs 3 and 4). From the mitral stack and commissural images, each scallop of the mitral valve leaflet (A1-P1, A2-P2, A3-P3) can be identified and any dysfunction defined (Fig 5). Phase-contrast through-plane velocity mapping of aortic and pulmonary artery flows are next performed using a previously described technique to allow quantification of the severity of mitral regurgitation [9]. We perform aortic velocity mapping in the ascending aorta immediately above the sino-tubular junction. In patients with ischemic mitral regurgitation, we further assess LV viability by inversion recovery late gadolinium imaging, and contractile reserve by low dose dobutamine stress at 5 and 10 mcg/kg/min as previously described [2,10]. The imaging protocol for assessing mitral regurgitation is summarised in the Appendix.

### Interpretation

**Figure 6:**
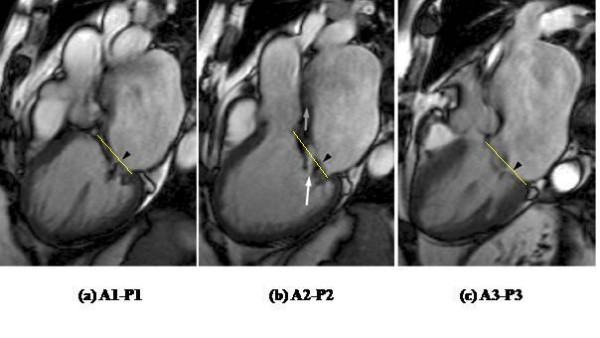
**Prolapse of P2 causing mitral regurgitation**. (a) A1-P1, (b) A2-P2, (c) A3-P3. Arrowhead shows the posterior leaflet. This can be seen to be prolapsed at (b) P2. The yellow line shows the mitral annular plane. The arrows show the eccentric jet of mitral regurgitation which results from failure of coaptation of A2-P2 and is anteriorly directed along the wall of the left atrium. The white arrow points to a central bright jet core in the A2-P2 bSSFP image, with a dark streak of signal loss beyond (grey arrow). This is in accord with relatively severe regurgitation.

The current recommendation for the assessment of mitral valve dysfunction as proposed by Carpentier and adopted by the American College of Cardiology focuses on the anatomic location and physiologic mechanism of the valve dysfunction and aims to help the surgeon in planning surgical repair of the valve [1,8]. Three types of mitral valve dysfunction are defined based on the maximal movement of the free edge of the leaflet during systole relative to the plane of the mitral annulus (the line joining the points of attachment of the valve leaflets to the annulus; Fig 6):

*Type I: normal leaflet motion *(annular dilatation, leaflet perforation).

*Type II: increased leaflet motion *(mitral valve prolapse, ruptured/elongated chordae or papillary muscle).

*Type IIIa: restricted leaflet motion in systole and diastole *(chordal or papillary muscle fusion or thickening from rheumatic valve disease).

*Type IIIb: restricted leaflet motion in systole *(functional mitral regurgitation due to tethering of mitral leaflet(s) from LV dysfunction and/or dilatation from myocardial infarction or non ischemic cardiomyopathy).

Type II dysfunction, as seen in mitral valve prolapse, is present when the free edge of the valve leaflet moves at least 2 mm above the plane of the mitral annulus during LV systole (Fig 6, see also additional file 1: Figure 6b movie). This results in an eccentric regurgitant jet directed away from the leaflet that is prolapsing. Posterior leaflet prolapse results in an anteriorly directed jet while anterior leaflet prolapse results in a posteriorly directed jet. Type III dysfunction is present when the normal or expected maximal leaflet movement is restricted. It is easily appreciated when severe as is typical in Type IIIa dysfunction due to rheumatic mitral valve disease. Less severe leaflet restriction occurring only during systole, as seen in Type IIIb dysfunction from myocardial infarction or cardiomyopathy, may be more difficult to appreciate by CMR unless one is familiar with the normal motion of the mitral valve leaflets and annulus. Measurements of the tethering distance from the mitral leaflet coaptation point to the mitral annulus plane can be helpful [11]. Leaflet restriction is suggested if no leaflet prolapse is identified despite the presence of an eccentric regurgitant jet. Leaflet restriction typically results in a regurgitant jet in the same direction as the leaflet restriction. For example, posterior leaflet restriction in ischemic mitral regurgitation usually results in a posteriorly directed regurgitant jet, while anterior leaflet restriction results in an anteriorly directed jet. If both leaflets are restricted, as is typical in dilated cardiomyopathy, a central jet of mitral regurgitation is more likely.

### Quantification of mitral regurgitation severity

Quantification of the severity of mitral regurgitation using CMR has been previously described [5-7]. The mitral regurgitant volume (MRV) is the difference between the LV stroke volume (LVSV) and the aortic forward stroke volume (AoSV) i.e. MRV (mls/beat) = LVSV – AoSV. The regurgitant fraction (RF) is the ratio of the MRV divided by the LVSV i.e. RF (%) = (MRV ÷ LVSV) × 100. This method of quantifying mitral regurgitation applies even in the presence of aortic regurgitation as long as only the systolic forward flow in the aorta is taken as the AoSV. Calculation of aortic flow using phase-contrast velocity flow mapping is well established but care must be taken to perform this optimally [9,12-14]. As a quality control, we ensure that the aortic forward flow is within 5% of the main pulmonary artery forward flow; the two values should be nearly equal in the absence of an intra-cardiac shunt. It may also be possible to directly measure mitral inflow volume by phase-contrast velocity flow mapping at the tips of the mitral valve leaflets but this requires a specialised imaging sequence which tracks the motion of the mitral valve annulus during the cardiac cycle [15].

In the absence of other regurgitant lesions, MRV can also be calculated by subtracting the right ventricle stroke volume (RVSV) from the LVSV i.e. MRV = LVSV – RVSV, using established techniques [3]. However, the calculation of RVSV is less reproducible compared to LVSV due to the extensive trabeculation of the right ventricle (RV). Moreover, associated tricuspid regurgitation is reported in up to 50% of patients with significant mitral regurgitation and this invalidates the use of RVSV to determine MRV [16].

The American College of Cardiology has established echocardiographic criteria for grading the severity of mitral regurgitation [1]. There are not yet established criteria for grading by CMR. However, regurgitant fractions (RF) calculated from CMR acquisitions have been correlated with echocardiographic grading in 83 patients with mitral regurgitation [5], although relatively few of these had more than moderate regurgitation. In the absence of established criteria for CMR, the findings of this study, derived from LV volume and ascending aortic flow measurements, can be noted: mild = RF ≤ 15%, moderate = RF 16–24%, moderate-severe = RF 25–42%, severe = RF > 42%.

### Qualitative grading of mitral regurgitation severity

Because the methods we suggest for quantifying mitral regurgitation by CMR rely on more than one set of measurements, each of which could contribute to inaccuracy if not optimally performed, it is important to support quantitative measurements with a qualitative visual assessment of severity. In our experience the contiguous stack of bSSFP cine acquisitions is well suited for this. Although the bSSFP sequences, with very short echo times, show only limited signal loss from turbulent regions, the non-velocity compensated sequence results in loss of signal from voxels located in the shear layers on either side of the core of high velocity jets. This is explained by dephasing of signal from voxels containing a wide range of velocities in the shear layer bounding a jet. In contrast to the dark shear layer voxels, bSSFP voxels lying within a coherent jet core, if present, show bright signal (Fig 6b, see also additional file 1: Figure 6b movie). For these reasons, suitably aligned bSSFP slices, either aligned with or transecting the jet, can show the width of any coherent jet core, which reflects the width of the regurgitant orifice (Appendix). Whereas the jets of mild mitral regurgitation will be too narrow to give bright central voxels, jet(s) of severe mitral regurgitation are likely to include jet core(s) either broad enough to give bright central voxels outlined by dark, or else extensive enough along the line of failed coaptation to be visible in several slices of the cine stack. In either case, a bSSFP cine aligned to transect the regurgitant jet core may also be valuable to assess the cross sectional extent of each jet. The principle illustrated in Fig 2 applies to the depiction of jet boundaries as well as leaflets. A further feature of severe mitral regurgitation is reversal of flow in the pulmonary veins during LV systole, which may be visible in the 4 chamber and certain mitral stack cines.

## Discussion

In this paper we have described the methods that we have used to assess mitral regurgitation by CMR in 149 patients over a period of 15 months. The etiologies of mitral regurgitation were varied with 42% due to ischemia or infarction, 41% degenerative, 9% due to rheumatic valve disease and 8% due to cardiomyopathy. We initially used this systematic imaging technique on a research study on ischemic mitral regurgitation (hence the large proportion of patients with ischemic mitral regurgitation), and found it to be informative and easy to perform, and now use it routinely. The methods described allow determination of the severity of mitral regurgitation, mitral valve dysfunction, LV volumes and function, and, when relevant, LV viability [2,3,5-7]. Evaluation of mitral valve dysfunction from standard, routinely acquired CMR imaging planes alone is rarely adequate. The technique we describe, with additional imaging of the mitral valve based on its anatomy, allows more detailed evaluation of its dysfunction. We have found such a systematic evaluation of the mitral valve to be valuable for all etiologies of valve dysfunction. In degenerative valve disease, it allows determination of the leaflet scallop responsible for the valve dysfunction e.g. P2 or P3 prolapse, and hence helps guide surgical repair. In rheumatic valve disease, it allows assessment of the severity of valve restriction and hence helps determine the feasibility of valve repair and the need for valve replacement. In functional mitral regurgitation due to ischemic heart disease or cardiomyopathy, it confirms the diagnosis and helps exclude coexisting degenerative valve disease. A proportion of patients with ischemic heart disease and mitral regurgitation have coexisting ischemic heart disease and degenerative mitral regurgitation, and not ischemic mitral regurgitation. It is important to distinguish between the two as functional ischemic mitral regurgitation may improve with only coronary revascularisation without a mitral valve repair but this would not be true for degenerative mitral regurgitation.

Comparison of the accuracy and reproducibility of CMR using this technique with echocardiography, especially transesophageal echocardiography, and findings at surgery will need to be done. Two recent studies using similar techniques as described here, but without the additional slices taken at the commissural ends of the mitral valve, have recently been published [17,18]. The first study reported a sensitivity and specificity of 89% and 88% respectively for detecting flail or prolapsed leaflets compared to findings at surgery in 47 patients. This compared with a sensitivity and specificity of 93% and 88% respectively for transesophageal echocardiography [17]. The second study reported agreement between CMR assessment and transthoracic echo determination of prolapsed or flail leaflets in 92% of 27 patients [18]. Although these studies involved only small numbers of patients, the results are encouraging given that the approach is new, and likely to improve with experience. We have found the additional slices acquired adjacent to the valve commissures to contribute to the visualisation of the valve leaflets in this region where, due to oblique relative orientation, they may be imperfectly seen in the mitral cine stack. These additional slices are useful in diagnosing A1/P1 or A3/P3 leaflet prolapse and also in diagnosing functional ischaemic mitral regurgitation where leaflet restriction is usually most marked at P3.

It is recognised that transesophageal echocardiography assesses the mechanisms of valve dysfunction well (leaflet prolapse/restriction) and is perhaps the technique best able to determine the structural lesion responsible for the incompetence (chordal/papillary muscle rupture/elongation, leaflet perforation, etc). The fixed imaging planes of CMR and its suboptimal through-plane resolution rarely permit adequate visualisation of the chordal structures to identify rupture or elongation accurately. CMR is also not suited for visualisation of leaflet and annular calcification which are important factors influencing the probability of successful valve repair. Transesophageal echocardiography, however, is operator dependent and is a semi-invasive investigation usually requiring patient sedation and may therefore be less desirable in some patients. Transthoracic echocardiography, although non-invasive, is also operator dependent and does not always provide adequate diagnostic images of valve dysfunction, particularly in patients with excess body mass. Localisation of valve defects to individual valve scallops can be difficult with transthoracic echocardiography. CMR, when used optimally, may therefore play a useful role in assessing the mitral valve in patients in whom transthoracic echocardiography has not provided adequate imaging and in whom transesophageal echocardiography is considered too invasive. It may also be useful to complement echocardiography in establishing the diagnosis when inadequately achieved by echocardiography. The accuracy of CMR in determining LV volumes, function and viability may be particularly useful.

## Conclusion

Comprehensive assessment of mitral regurgitation requires assessment of: (1) its severity to determine the need for surgical intervention (mild/moderate/severe: MRV and RF); (2) the mechanism of the dysfunction to determine the type of surgical intervention required (leaflet prolapse/restriction, including the leaflet scallops involved: A1-P1, A2-P2, A3-P3); (3) LV volumes and function to determine the timing and risks of surgery; and, in ischemic mitral regurgitation, (4) LV viability. Such comprehensive assessment is feasible in a single CMR examination but needs a defined protocol, as described in this paper. When used optimally, CMR can complement existing imaging modalities such as echocardiography in the assessment of patients with mitral regurgitation.

## Abbreviations

AoSV: Aortic forward stroke volume; bSSFP: Balanced steady state free precession; CMR: Cardiovascular magnetic resonance; LV: Left ventricle; LVOT: Left ventricular outflow tract; LVSV: Left ventricular stroke volume; MRV: Mitral regurgitant volume; RF: Regurgitant fraction; RVOT: Right ventricular outflow tract; RVSV: Right ventricular stroke volume.

## Competing interests

The authors declare that they have no competing interests.

## Authors' contributions

KMJC, JRP, DJP and PJK conceived the project. KMJC and PJK developed the imaging protocol and wrote the first and final drafts of the manuscript. RW and KS helped develop and optimise the imaging protocol and performed the imaging scans. DNF helped develop and optimise the imaging protocol. All authors provided scientific contributions, participated in the editing of the manuscript, and read and approved the final draft.

## Appendix

**Recommended CMR protocol for the assessment of mitral regurgitation**.

(1) **Scout images**, including transaxial and coronal multislice stacks.

(2) **Standard ventricular cines acquisitions **using bSSFP:

• Vertical long axis (2 chamber)

• Basal short axis scout cine(s)

• Four chamber, aligned through LV apex and the centres of mitral and tricuspid valves.

• LVOT (3 chamber) and coronal LVOT cross cut cine

• Aortic valve cine, in the plane of aortic valve coaptation

• Oblique sagittal RVOT cine

• Short axis cine stack for ventricular volume measurement

(3) **Mitral cine stack **using bSSFP:

5 mm slice thickness with no inter-slice gaps. The orientation is perpendicular to the central part of the line of mitral coaptation and approximately parallel to the LVOT or 3 chamber cine. After alignment, the first slice is placed through the most superior mitral commissural point, as seen in basal short axis cines. The remaining 8–10 slices are acquired sequentially through A1-P1, A2-P2, and A3-P3 mitral scallops, continuing to just beyond the more inferior commissure.

(4) **Additional commissural cines **are acquired perpendicular to the lines of coaptation adjacent to each of the commissures, if these are oblique to the central coaptation line.

(5) **Cine(s) aligned to transect each mitral regurgitant jet**, located immediately atrial of the regurgitant orifice. Spatial resolution should be optimised. This approach can help to demonstrate severity in occasional cases where there is a discrete and coherent regurgitant jet core. Through-plane velocity mapping, with appropriate VENC, may also be used, although the spatial resolution is likely to be suboptimal.

(6) **Through-plane velocity mapping of aortic and pulmonary flow**, VENC as low as possible without aliasing (typically 150 cm/s, but more if necessary).

Additional, if indicated in ischemic mitral regurgitation:

(7) **Late gadolinium enhancement **inversion recovery imaging to determine myocardial viability.

(8) **Low dose dobutamine cine imaging **to assess contractile reserve.

**Coronary angiography **using a fat suppressed 3-D bSSFP diaphragm navigator acquisition, if indicated, to visualise the proximal coronary arterial courses.

## Supplementary Material

Additional file 1 Figure 6b (movie). bSSFP cine demonstrating prolapsed P2 with eccentric jet of severe mitral regurgitation directed anteriorly along the wall of the left atrium and extending to its back wall. A central bright jet core is seen with a dark streak of signal loss beyond indicating severe regurgitation. Click here for file
